# Improving Self-Efficacy, Quality of Life, and Glycemic Control in Adolescents With Type 1 Diabetes: Randomized Controlled Trial for the Evaluation of the Family-Centered Empowerment Model

**DOI:** 10.2196/64463

**Published:** 2024-12-10

**Authors:** Salah Alzawahreh, Candan Ozturk

**Affiliations:** 1 Ministry of Health Amman Jordan; 2 Faculty of Nursing Near East University Cyprus Turkey

**Keywords:** adolescents, family-centered empowerment model, glycemic control, quality of life, self-efficacy, type 1 diabetes mellitus, T1DM, family-centered, teenager, glycemic, experimental evaluation, empowerment, Jordan, glycosylated hemoglobin, HbA1c, experimental study, family, care education, self-care, educational program, mobile phone

## Abstract

**Background:**

Poor glycemic management in adolescents with type 1 diabetes mellitus (T1DM) increases complications. Enhanced control is associated with other factors, such as cultural, socioeconomic, and health care system disparities specific to the Middle East, which can greatly influence individuals’ ability to get and use health care services as well as their reaction to treatment approaches.

**Objective:**

This study aims to evaluate the impact of the family-centered empowerment model on Jordanian adolescents with T1DM, focusing on their glycosylated hemoglobin levels, self-efficacy, and quality of life (QOL).

**Methods:**

A randomized controlled trial involved 68 adolescents with T1DM visiting Jordanian Royal Medical Services’ clinics. Two sets of participant groups were created: control (n=34) and intervention (n=34). Participants were randomly assigned to either the intervention group, receiving the family-centered empowerment model intervention, or the control group, receiving standard care. Data were collected through face-to-face interviews and medical records.

**Results:**

From April to October 2023, a total of 68 adolescents with T1DM participated in the study at the Jordanian Royal Medical Services. QOL had significant improvement among 13 (38%) of the 34 participants in the intervention group, and the program significantly improved moderate self-efficacy levels in 12 (35%) patients (*P*<.001). In addition, the average glycosylated hemoglobin levels dropped from 11.25% to 10.23% (*P*<.001). Additionally, improvements were seen in stress management, communication, and treatment adherence, with a substantial decrease in treatment obstacles. The intervention was successful in improving both clinical and psychosocial outcomes, as evidenced by the fact that the control group showed no noticeable improvements in these parameters.

**Conclusions:**

The study suggests that patients with T1DM should receive continuous care education sessions, including self-care training, to improve their health. Nurses should also incorporate this training into treatment plans and educational programs for adolescents to enhance their QOL.

**Trial Registration:**

ClinicalTrials.gov NCT06694467; https://clinicaltrials.gov/study/NCT06694467

## Introduction

Diabetes mellitus is a prevalent and increasingly acknowledged medical disorder that affects individuals worldwide. It is a metabolic condition marked by elevated blood glucose levels that can cause serious side effects like renal failure, heart disease, blindness, and even amputations [1]. For type 1 diabetes mellitus (T1DM), to keep their blood glucose levels within a target range and avoid problems, patients must take insulin therapy for the rest of their lives and exercise cautiously [2]. The essentials of managing T1DM are insulin delivery, carbohydrate counting, and blood glucose monitoring [2]. The occurrence of T1DM at regional and population levels is of great interest, indicating that geographic location and ethnicity can have a significant impact on the prevalence of T1DM [3,4]. Worldwide distribution of this illness appears to be influenced by both genetic and environmental variables, while the reasons for these variances are still a mystery to experts [4].

T1DM is a challenging disease that requires proper adherence to treatment by adolescents, who otherwise will experience adverse health issues in their management. These health issues are further compounded by the unsatisfactory glycemic control. Some of the cultural aspects applicable to the Middle East include perception on health and diabetes and family dynamics, which are the patterns of relationships and interactions within a family, including responsibilities, communication techniques, emotional bonding, and cultural influences, which may have an impact on glucose control. A child’s attitude and strategy for treating their diabetes may be greatly impacted by these dynamics. Children with T1DM often depend on their families’ support and encouragement to follow their treatment plans, dietary recommendations, and daily physical activity [5,6]. The American Diabetes Association recommends parents and caregivers to educate themselves about T1DM; attend diabetes education classes; and learn insulin administration, blood glucose interpretation, hypoglycemia or hyperglycemia signs, ketoacidosis treatment, and carbohydrate counting [7].

Previous studies concerning family-centered practices have revealed the curiosity of integrating families in the treatment process of several conditions such as T1DM [8]. The teamwork coping skills intervention is one of the most popular works that was conducted by Harris et al [9]. The purpose of this study was to improve coping skills among the population with T1DM by increasing teamwork between adolescents and the members of their households. This paper showed that families can coalesce into a productive unit that not only improves the clinical status of the youngster with diabetes but also encourages caregivers to provide more emotional support to adolescents with the disease. Harris et al [9] stated that family context is a critical component that contributes to the effectiveness of diabetes management programs. The finding suggests that the provision of coping skills to families, along with supportive communication around treatment, empowers adolescents to feel more supported and thus largely enhances their self-efficacy and treatment enactment. This study relates with the objectives of the present research that focuses on family-based empowerment to enhance the health status of Jordanian adolescents with T1DM. One possibility of results of this type of study indicates that the use of family-focused models in diabetes treatment may not only improve clinical measures, including glycosylated hemoglobin (HbA_1c_), but also dramatically influence psychosocial health. Family-centered interventions are medical strategies that involve family members in the management and treatment of a patient’s disease. Increased adherence to medical advice is one of the many advantages that these therapies have been documented to provide [8]. Furthermore, they can help to reduce parental anxiety as well as to facilitate better communication between the health care team and the family [8]. Harris et al [9] established the Novel Interventions in Children’s Healthcare initiative to help adolescents with chronic illnesses, such as T1DM, who struggle to manage their illness. To improve family dynamics, Novel Interventions in Children’s Healthcare offers comprehensive case management, individualized behavioral treatments, and care coordination between family members and medical professionals. By addressing adherence hurdles brought on by outside variables including housing, insurance, and educational institutions, this strategy seeks to enhance overall illness management and health outcomes [9].

Adolescents who have greater levels of self-efficacy also tend to have higher levels of self-confidence and positive self-esteem. For people with diabetes, physical health plays a significant role in determining quality of life (QOL) [10,11]. While parental involvement is known to affect adolescent health in a grand way, it is argued that the inclusion of parents’ feedback would complement the studies and present a diverse opinion about the effectiveness of the intervention [12]. Additionally, if the researchers inquired how the parents adjusted to or how they helped the children during the intervention, it will give an insight into what kind of family scenarios were in play. For example, parents could give feedback regarding increased use of communication, changes in their or their child’s role in diabetes management, or changes in attitude toward diabetes care [13]. The objective of this randomized clinical trial is to evaluate how the family-centered empowerment model (FCEM) affects the clinical and psychological outcomes of adolescents with T1DM, with an emphasis on increases in QOL, self-efficacy, and HbA_1c_ levels.

## Methods

### Study Design and Settings

A randomized controlled clinical trial was conducted from April to October 2023 at the Jordanian Royal Medical Services in Amman, Jordan, and 68 participants were gathered by October 1, 2023. Analysis of the data was completed on November 15, 2023. The study was reported in accordance with the CONSORT (Consolidated Standards of Reporting Trials) statement (Multimedia Appendix 1).

### Study Population

#### Patients and Recruitment

This clinical trial involved individuals with T1DM, aged 12-18 years, who had not taken part in any professional diabetes education program for the preceding month of the study and who had been diagnosed at least 6 months earlier. According to the direction of medical professionals, the course discussed topics such as diabetes types, blood sugar regulation, self-monitoring, exercise, diet, medicine administration, and problem-solving methods, which frequently include psychosocial support. The study excluded adolescents who had severe long-term diseases or newly diagnosed T1DM.

An official diabetes education program is a planned educational endeavor designed to provide people with diabetes and their families the information and abilities they need to manage their condition effectively. These parameters were adopted to ensure that the study takes place in a controlled environment and targets the aspects of intervention of the study. To ensure a representative sample from the clinic, many recruitment techniques were used for this study. In the waiting rooms, we distributed brochures and announcements with information. The recruitment of adolescents with T1DM for this study involved invited participants with T1DM attending the Jordanian Royal Medical Services clinics. Overall, 110 patients were assessed for eligibility; of them, 68 adolescents fulfilled the aforementioned inclusion criteria. After being recruited, the study design and objectives were illustrated to the patients and their families, and written informed consent was obtained. The participants were given anonymous serial numbers, which were randomized using an electronic randomizing app, Research Randomizer (version 4.0; Social Psychology Network). The sample was representative of the clinic’s demographics, including age, sex, and socioeconomic status, according to a comparison of the clinical and demographic characteristics of the recruited participants with the clinic’s larger patient population. Patients were followed up for a period of 6 months. There was no loss of follow-up, and none of the enrolled patients withdrew from the study. With respect to the study population, the mean duration of diabetes was 4.69 (SD 3.3) years for the intervention group and 3.581 (SD 2.2) years for the control group. Pen-based insulin administration systems were used, using rapid-acting and long-acting formulations. Traditional glucometer readings were used to regulate glycemia without continuous glucose monitoring. The intervention and control groups were derived from a combination of evidence-based practices gleaned from previous similar studies, pilot studies, and clinical experiences [14].

#### FCEM Intervention

The FCEM intervention aimed to help engage the adolescent, an individual with T1DM, and his or her family in the self-management process. The intervention consisted of several key components: first, education sessions: over the course of the study, the participants received educational training in various aspects of diabetes such as diet, exercise, glucose monitoring, and insulin administration. Emotional and psychological needs also formed an essential component of the curriculum that was discussed. Cognitive behavioral therapy was provided in group sessions, which was conducted over 4 weeks. Other materials used included brochures, videos, and brainstorming activities. Second, goal-setting workshops: these workshops stimulated participants to come up with sound, reasonable, and achievable personal health goals in regard to their condition of diabetes. Third, family involvement: parenting skills were offered to enable families to attend sessions in order to help create a support base for them at home. It was noted from discussions concerning diabetes self-management that changes in family functions have a close relationship with diabetes self-management. The other focus included a discussion of common issues that families experience and deal with. Fourth, psychosocial support: the intervention included counseling services, including stress management and communication with health care providers and how to live with chronic health conditions. While engaging families in the educational process and treating for psychosocial needs, we sought to enhance the supportive environment that may lead to better health for adolescents with T1DM. Additionally, joint educational workshops were conducted, in which parents engaged in educational programs together with the adolescents. These workshops included general information on T1DM, insulin, food, exercise, and stress management. Parents were also able to learn on how they could help their children in case they encountered problems by attending these classes together [15]. Furthermore, parents can do interactive role-playing activities where the intervention involved hypothetical situations in which adolescents and parents could practice their communication skills. Parents got knowledge on how to deal with health care providers and assist their adolescents during planning for management approaches [16]. In addition, they were trained on family goal-setting, where the families were supposed to set realistic health-related goals on their own in order to work as a team in achieving diabetes management goals. Moreover, support groups were created, and parents were encouraged to join the groups where they exchanged their experiences especially the challenges they faced while parenting a child with T1DM. They brought in a support group and gave parents tools on how to deal with their children together with an opportunity to interact with fellow parents [17]. Regular follow-up communication was conducted after the course of the intervention. This included telephone calls and SMS text messages to inform them of their child’s progress, and additional information given to demonstrate what is taught in sessions. A feedback session was also conducted to help in further enhancing the internal validity; parents were afforded the chance to give input as to the content and presentation of the intervention. This way, they used their previous experiences to provide more appropriate content to the program to suit the needs of the adolescents and their families. Finally, at the end of the intervention, parents were involved in talks that elaborated an assessment of the program and the result achieved [18].

#### Research Instrument

In this study, a 4-part research instrument was used (Multimedia Appendices 2-4). The first section of the questionnaire was sociodemographic in nature, covering topics such as age, sex, school grade, age at diagnosis, educational background and employment of the parents, length of illness, number of daily injections, frequency of daily blood glucose checks, number of diabetes-related hospitalizations last year, and episodes of hypoglycemia reported last month. HbA_1c_ readings were used to monitor glucose management in teenagers, following the American Diabetes Association’s recommendation for values below 7% using Bio-Rad’s high-performance liquid chromatography technology. The Self-Efficacy Questionnaire (SEQ), which had 3 subscales, was the second portion. The subscales were academic self-efficacy (items 1, 4, 7, 10, 13, 16, 19, and 22), social self-efficacy (items 2, 6, 8, 11, 14, 17, 20, and 23), and emotional self-efficacy (items 3, 5, 9, 12, 15, 18, 21, and 24). In total, 8 components make up each subscale. The entire instrument consisted of 24 items, and each item was evaluated on a 5-point Likert scale (from 1 to 5), with a possible score range of 24-120 [19]. A high score denotes a high degree of self-efficacy. The self-efficacy levels were therefore classified as low (less than 60%), moderate (between 60% and 80%), and high (more than 80%). The third section consisted of a questionnaire called the Pediatric Quality of Life Diabetes Module. The 28 items evaluated on a 5-point Likert scale (from 0 to 4) in this multidimensional tool were categorized into 5 domains: treatment obstacles (4 items), treatment adherence (7 items), concern (3 items), diabetic symptoms (11 items), and communication (3 items). For teenage self-report, all of the elements were flipped and linearly translated to a 0-100 scale in order to calculate each score. The total score was computed by adding all items on the whole scale and dividing by the total number of answered items [20]. Given the total score range from 0 to 112, we classified the scores as low (0 to 37), moderate (38 to 74), and high (75 to 112). Higher scores corresponded to improved QOL. Medical records pertaining to the glucose control data comprised the fourth section. In the present context, the reliability and validity of the measures are worthy of discussion.

To determine reliability, the study used standardized questionnaires that have been used in other studies with the intention of estimating self-efficacy, HbA_1c_, and QOL. Regarding HbA_1c_, blood samples were tested based on specific time-stable laboratory techniques. Self-efficacy was measured with a reliable instrument developed and standardized for the adolescent population in a previous study, and SEQs used had high internal consistency (Cronbach α>0.85). The QOL was measured by reliable and internationally used instruments, such as the Pediatric Quality of Life Inventory, and tools were validated beforehand in similar samples.

The internal validity of the measures was tested by means of content and construct validation. With regard to the instruments, those used in this study were relevant to the culture of the Jordanian population. To increase confidence in the measures, the researchers used pilot testing with a sample of adolescents prior to the main study to determine whether the measures properly indexed the intended constructs. Evaluations were performed for several months from April through October 2023 in order to capture changes to measures for the formal and informal sectors. Subsequently, the follow-up assessments were planned to reveal the acute rather than long-term effects of the intervention. The data processing was done before November 15, 2023. Based on the concept, FCEM was put into practice over the course of 4 weeks, with 4 sessions. Adolescents and their families were arranged in a teaching group using smartphone apps prior to each session, and the lessons were conducted via sharing instructional materials and a video. All participants and their families participated in phone group sessions.

For research samples in the case group, the 4 phases of the empowerment model, perceived danger, problem-solving, instructional involvement, and evaluation, were used to apply its contents. Perceived threat, which is comprised of perceived severity and perceived vulnerability, is the first stage. “Perceived severity” refers to the degree to which a person and their family recognize the dangers or difficulties associated with a sickness and believe that a condition is possible. The researcher intends to comprehend the issue using the study samples, provide answers, and put them into practice in the second stage, which is to increase self-efficacy. The third step constituted of boosting self-esteem through instruction participation, as patients were given instructions on healthy lifestyle and were able to participate with the group to implement these instructions. It is noteworthy to mention that instruction and participation were also consistently provided in the first and second phases. Process and summative evaluations were part of the fourth step. During the process assessment, every session was assessed to guarantee the patient’s subjective and practical involvement in the care plan and to confirm that they are adhering to the previously given instructions [21]. The control group did not get any intervention in this area and just got standard treatment in the diabetic clinic.

### Data Analysis

The data review was conducted using SPSS (version 22; IBM Corp). Initially, any necessary adjustments were performed once the data were examined for data entry problems. All enrolled patients were included in the statistical analysis (Multimedia Appendix 5). For descriptive data, the variables mean and SD or number and percentage were used; steepness and skewness were assessed in relation to the scale data’s normal distribution. The frequency distribution was used for categorical variables, while the mean and SD were used for continuous variables. The test known as the 2-tailed *t* test was used for comparing means between a pair of continuous variables, while the chi-square test was used to look at the connection between 2 categorical variables. Furthermore, the 2 independent sample 2-tailed *t* test was used to examine if there was a statistically significant difference between the experimental and control groups. We evaluated the relationship between glycemic control, SEQ, and QOL using the Pearson correlation coefficient. A *P* value of less than .05 indicated that the results were significant.

### Ethical Considerations

The study protocol was approved by the institutional review board committee of the Near East University on January 26, 2023 (acceptance NEU/2023/110-1681). This trial was registered on ClinicalTrials.gov (NCT06694467). Adolescents and their families received complete details about the study’s goals, methods, risks, and benefits before giving their informed permission. The recruitment process maintained the privacy of participants, promoting an open and ethical research setting, and all the collected data were anonymized and used solely for statistical analysis. As a measure of respecting the research subjects, participants and their guardians provided their written informed consent (Multimedia Appendix 6). Informed consent was first given by 12- to 18-year-old adolescents regarding the aims and overall procedures of the study and possible risks and benefits of participation. Additionally, the parents or the guardians and the adolescents were allowed to ask questions in order to be clear on the aspects involving their participation. We explained that the participation was voluntary, and the participant could withdraw from the study with any consequences to health care. Compensation was not provided in order not to affect participants’ motivation. The consent process also played a crucial role in ensuring and gaining the confidence of the families, meaning reliable data were collected. Consistent with the principles of procedural protectiveness, this comprehensive procedural plan corresponds with the family-centered intervention literature, indicating that a strong foundation of ethical practice is key to intervention completion and participant response. With this information included, the study can adequately discuss the ethical considerations that are present in research involving adolescents and stress that involving families may help to improve the care of adolescents with T1DM.

## Results

### Recruitment of Participants

**Figure 1:**
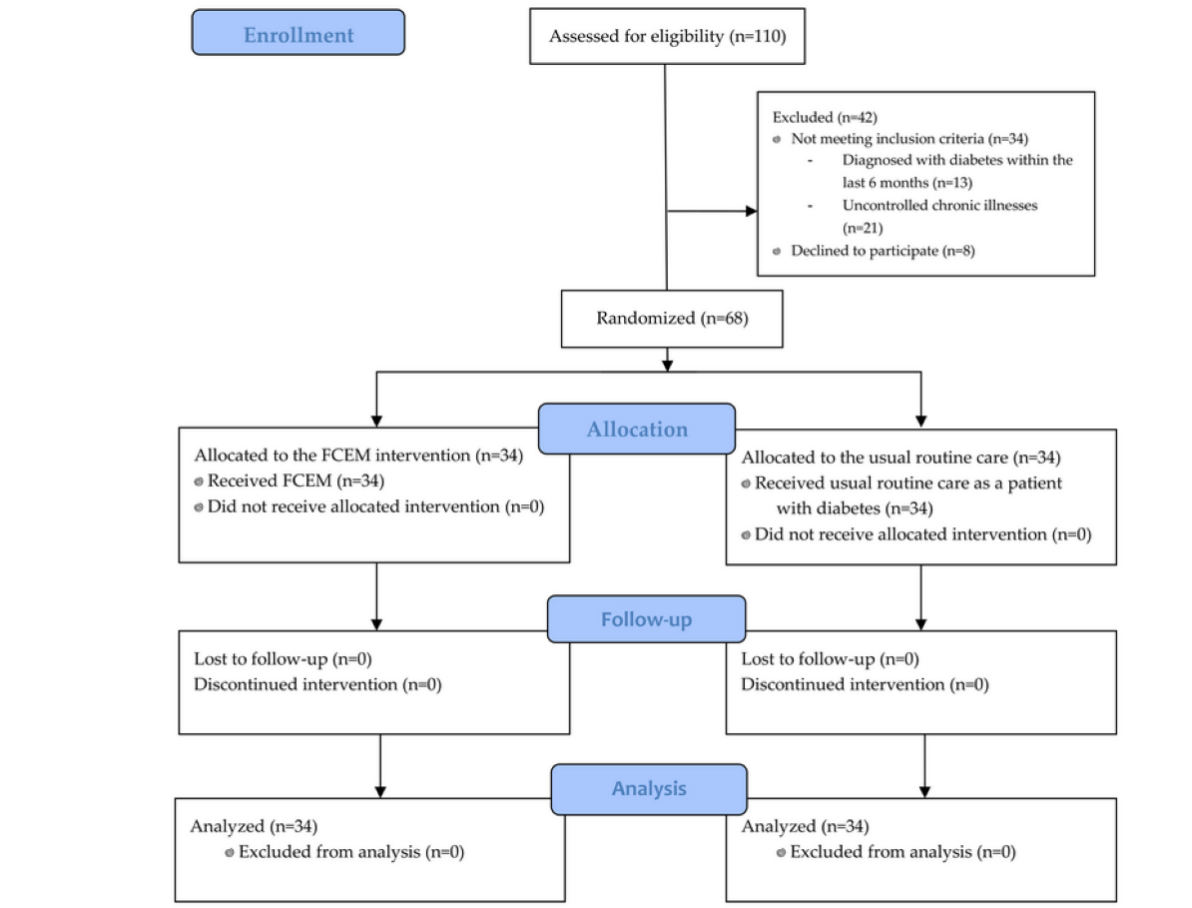
The CONSORT (Consolidated Standards of Reporting Trials) flowchart of study participants’ enrollment. FCEM: family-centered empowerment model.

This study included 68 teenagers with T1DM (Figure 1). Age (*P*<.001), weight (*P*=.01), and student level (*P*<.001) were the 3 categories in which there was a significant difference in the groups’ mean scores. The mean age of participants in the intervention group was 15.1 (SD 1.4) years, which was substantially older than the control group’s mean age of 13.3 (SD 1.6) years (*P*<.001). The mean weight in the intervention group was 57.9 (SD 12.1) kg, which was significantly higher compared to the control group, with a mean weight of 48.7 (SD 14.5) kg (*P*=.01). In total, 10 (29%) out of 34 participants were affected by diabetes in the control group and 12 (35%) out of 34 participants had diabetes in the intervention group; however, this difference was not statistically significant.

Based on maternal and paternal educational attainment, insurance, height, sex, and other factors, the statistical test findings revealed no statistically significant difference between the 2 groups (all *P*>.05). In addition, it shows that insulin injections were administered 3 times a day to both groups of patients with T1DM, and most of the patients had T1DM for 5 years or more with no appreciable changes. In total, 9 (26%) of 34 patients in the intervention group and 2 (6%) of 34 patients in the control group had a first-degree family history of diabetes mellitus; however, most members of the 2 groups had just 1 hospitalization (Table 1).

**Table 1:** Comparison of the participant demographics and participant characteristics linked to diabetes in the intervention and control groups (N=68).

Variable		Intervention group (n=34)	Control group (n=34)	*P* value	
**Age (years), mean (SD)**		15.1 (1.4)	13.3 (1.6)	<.001	
**Sex, n (%)**					.15
	Female	20 (59)	14 (41)		
	Male	14 (41)	20 (59)		
**Weight (kg), mean (SD)**		57.9 (12.1)	48.7 (14.5)	.01	
**Height (cm), mean (SD)**		158.8 (9.9)	153.8 (12.3)	.07	
**Student level (grade), mean (SD)**		9.5 (1.5)	7.5 (1.8)	<.001	
	≤8th grade, n (%)	8 (23)	27 (79)	<.001	
	>8th grade, n (%)	26 (77)	7 (21)	<.001	
**Educational level of father, n (%)**					.67
	Less than high school	6 (17)	6 (18)		
	High school degree or equivalent	16 (47)	18 (53)		
	College degree	3 (9)	3 (9)		
	Bachelor’s degree	7 (21)	6 (18)		
	Master’s degree	0 (0)	1 (3)		
	Doctoral degree	2 (6)	0 (0)		
**Educational level of mother, n (%)**					.43
	Less than high school	8 (23)	9 (27)		
	High school degree or equivalent	13 (38)	13 (38)		
	College degree	7 (21)	3 (9)		
	Bachelor’s degree	5 (15)	9 (26)		
	Master’s degree	1 (3)	0 (0)		
	Doctoral degree	0 (0)	0 (0)		
**Do you live with both of them, n (%)**					.55
	Yes	33 (97)	32 (94)		
	No	1 (3)	2 (6)		
**Insurance (yes), n (%)**		33 (97)	34 (100)	.31	
**Age at diagnosis (years), mean (SD)**		10.4 (3)	9.5 (2)	.18	
**Duration of diabetes (years), n (%)**					.07
	1 to <2	12 (35)	10 (29)		
	2 to <3	1 (3)	9 (27)		
	3 to <4	1 (3)	2 (6)		
	4 to <5	5 (15)	4 (12)		
	>5	15 (44)	9 (26)		
**Do any members of your family have a history of diabetes? n (%)**					.02
	Yes	9 (2)	2 (6)		
	No	25 (74)	32 (94)		
**Injections per day, mean (SD)**		3.1 (0.6)	3.0 (0.4)	.60	
**How often is a daily blood sugar test performed? mean (SD)**		2.6 (1.2)	3.5 (2.1)	.05	
	Once, n (%)	6 (17)	9 (26)	.01	
	Twice, n (%)	5 (15)	0 (0.0)	.01	
	Three or more, n (%)	16 (47)	24 (71)	.01	
	Never, n (%)	7 (21)	1 (3)	.01	
**How many times did diabetes keep you in the hospital last year? mean (SD)**		1.5 (1.6)	1.3 (1.1)	.44	
	Once, n (%)	13 (38)	18 (53)	.65	
	Twice, n (%)	5 (15)	3 (9)	.65	
	Three or more, n (%)	7 (21)	6 (17)	.65	
	Never, n (%)	9 (26)	7 (21)	.65	
**How many episodes of hypoglycemia have you reported last month, mean (SD)**		2.3 (2.5)	2.2 (2.2)	.84	
	Once, n (%)	5 (15)	3 (9)	.23	
	Twice, n (%)	7 (21)	2 (6)	.23	
	Three or more, n (%)	12 (35)	15 (44)	.23	
	Never, n (%)	10 (29)	14 (41)	.23	

### Diabetes Self-Efficacy Scores Across Program Phases in Intervention Group

Table 2 shows that every component of self-efficacy in the intervention group (n=34) significantly improved from before to after the therapy session. The mean scores of social, emotional, and academic self-efficacy all rose from 26.91 (SD 4.8) to 30.50 (SD 3.5; *P*<.001), from 28.85 (SD 3.9) to 34.47 (SD 11.0; *P*=.04), and from 25.76 (SD 2.9) to 30.11 (SD 5.3; *P*<.001), respectively. The mean overall self-efficacy increased from 81.53 (SD 10.3) to 95.09 (SD 14.9; *P*<.001), indicating a substantial improvement.

**Table 2:** Mean diabetes adolescent self-efficacy scores throughout the educational program phases among intervention group (n=34)^a^.

Domains of self-efficacy in adolescents	Intervention group (n=34)		*P* value	Control group (n=34)		*P* value
	Preprogram, mean (SD)	Postprogram, mean (SD)		Preprogram, mean (SD)	Postprogram, mean (SD)	
Academic self-efficacy	28.85 (3.9)	34.47 (11.0)	.04	28.35 (3.1)	28.53 (5.6)	.85
Social self-efficacy	26.91 (4.8)	30.50 (3.5)	<.001	23.26 (3.3)	26.0 (8.4)	.06
Emotional self-efficacy	25.76 (2.9)	30.11 (5.3)	<.001	21.88 (3.6)	22.03 (6.9)	.90
Total	81.53 (10.3)	95.09 (14.9)	<.001	73.50 (9.1)	71.55 (8.6)	<.001

^a^Mean self-efficacy score was calculated using the Self-Efficacy Questionnaire, which had 3 subscales: academic self-efficacy, social self-efficacy, and emotional self-efficacy. In total, 8 components make up each subscale, each being evaluated using a 5-point Likert scale, with a possible score range of 8-40 per each subscale and 24-120 for the total score.

On the other hand, the control group (n=34) showed minimal changes. The mean score of social self-efficacy went from 23.26 (SD 3.3) to 26.0 (SD 8.4; *P*=.06), emotional self-efficacy went from 21.88 (SD 3.6) to 22.03 (SD 6.9; *P*=.90), and academic self-efficacy went from 28.35 (SD 3.1) to 28.53 (SD 5.6; *P*=.85). When compared to mean 73.50 (SD 9.1) before the program, total self-efficacy decreased to mean 71.55 (SD 8.6; *P*<.001). Among intervention participants, the program significantly improved moderate self-efficacy in 12 (35%) of 34 patients while decreasing low self-efficacy in 8 (23%) of 34 patients. Nevertheless, it had no effect on the high levels of self-efficacy that 14 (42%) of 34 patients maintained throughout the program.

### QOL Scores Across Program Stages

When comparing the postprogram follow-up evaluation to the preprogram phase, Table 3 shows that adolescents in the intervention group (n=34) showed significant improvements in every aspect of their QOL from before to after the program. The mean diabetes symptom scores rose from 32.82 (SD 12.0) to 40.44 (SD 10.7; *P*<.001), treatment barriers scores improved from 55.88 (SD 13.3) to 71.69 (SD 12.6; *P*<.001), treatment adherence scores rose from 63.65 (SD 15.9) to 77.31 (SD 10.5; *P*<.001), diabetes-related stress scores increased from 44.85 (SD 21.2) to 60.78 (SD 24.0; *P*<.001), and communication score improved from 73.04 (SD 26.3) to 82.35 (SD 20.5; *P*<.001). There had been small or no differences in the control group (n=34). The mean treatment barriers scores changed from 64.34 (SD 13.0) to 62.31 (SD 10.9; *P*=.12), treatment adherence scores dropped from 68.48 (SD 2.7) to 66.28 (SD 9.7; *P*<.001), diabetes-related stress scores dropped from 49.51 (SD 18.6) to 43.31 (SD 19.4; *P*<.001), and communication scores dropped from 75.24 (SD 13.8) to 66.67 (SD 14.4; *P*<.001).

**Table 3:** Mean quality of life scores for patients with diabetes throughout the educational program stages among intervention group (n=34)^a^.

Domains of quality of life in adolescents	Intervention group (n=34)			*P* value		Control group (n=34)			*P* value
	Preprogram, mean (SD)	Postprogram, mean (SD)			Preprogram, mean (SD)		Postprogram, mean (SD)		
Diabetes symptoms	32.82 (12.0)	40.44 (10.7)	<.001		35.05 (9.7)		33.61 (9.8)	.05	
Treatment barriers	55.88 (13.3)	71.69 (12.6)	<.001		64.34 (13.0)		62.31 (10.9)	.12	
Treatment adherence	63.65 (15.9)	77.31 (10.5)	<.001		68.48 (2.7)		66.28 (9.7)	<.001	
The worry about diabetes	44.85 (21.2)	60.78 (24.0)	<.001		49.51 (18.6)		43.31 (19.4)	<.001	
Communication	73.04 (26.3)	82.35 (20.5)	<.001		75.24 (13.8)		66.67 (14.4)	<.001	

^a^The 28 items for the quality of life questionnaire were categorized into 5 domains: treatment obstacles (4 items), treatment adherence (7 items), concern (3 items), diabetic symptoms (11 items), and communication (3 items). For teenage self-report, a 5-point Likert scale (from 0 to 4) was used, and total scores varied from 0 to 112.

The results demonstrate that 13 (38%) of 34 patients who got the intervention following the educational session had significant improvements in their QOL. The percentage of participants reporting low QOL decreased substantially (10/34, 29%), whereas the percentages reporting moderate and good QOL increased noticeably (11/34, 32%).

### HbA1c Score Comparison Between Intervention and Control Groups Before and After the Intervention

As shown in Table 4, HbA_1c_ readings in the intervention group (n=34) showed a significant improvement, with a mean difference of –1.02% (SD 0.8%) from a mean of 11.25% (SD 2.4%) at the first visit to 10.23% (SD 2.1%) after 6 months (paired *t* test: t_33_=7.43; *P*<.001). With a mean difference of 0.63% (SD 0.5%), the control group (n=34) showed a statistically significant decline from their baseline mean HbA_1c_ of 9.58% (SD 1.5%) to 10.21% (SD 1.5%) at 6 months (paired *t* test: t_33_=–6.80; *P*<.001). The 2 groups’ HbA_1c_ changes over time were significantly different, according to an independent 2-tailed *t* test, with the intervention group showing a larger improvement (mean difference between groups=–0.39; t_65_=–9.91; *P*<.001).

**Table 4:** Comparison of mean glycosylated hemoglobin (HbA1c) scores before and after intervention, both within and across intervention and control groups.

HbA_1c_	Intervention group (n=34), mean (SD)	Control group (n=34), mean (SD)	Independent *t* test (*df*)
First visit	11.25 (2.4)	9.58 (1.5)	7.43 (33)^a^
6 months	10.23 (2.1)	10.21 (1.5)	–6.80 (33)^a^
Mean difference	–1.02 (0.8)	0.63 (0.5)	–9.91 (65)^a^
Paired *t* test (*df*)	7.43 (33)^a^	–6.80 (33)^a^	N/A^b^

^a^*P*<.001.

^b^N/A: not applicable.

There is a strong positive correlation between self-efficacy levels before and after the test among intervention (*P*<.001), whereas there is a negative correlation between HbA_1c_ and SEQ levels after the test without significant values. In addition, there is a strong positive correlation between QOL-pretest and QOL-posttest (*P*<.001; Table 5).

**Table 5:** Pearson correlation (r) between glycosylated hemoglobin (HbA1c), quality of life (QOL), and self-efficacy levels among intervention and control groups.

	Intervention group, *r*	*P* value	Control group, *r*	*P* value
SEQ^a^-pretest versus SEQ-posttest	0.772	<.001	0.942	<.001
QOL-pretest versus QOL-posttest	0.651	<.001	0.846	<.001
HbA_1c_-last versus SEQ-posttest	–0.010	.96	0.224	.21
HbA_1c_-last versus QOL-posttest	0.158	.37	–0.410	.02

^a^SEQ: self-efficacy questionnaire.

## Discussion

### Principal Findings

The study’s main findings showed that adolescents with T1DM experienced markedly better clinical and psychosocial results while using the FCEM. Significant improvements in intellectual, social, and emotional self-efficacy were observed in the intervention group (*P*<.001), with a 35.3% rise in moderate self-efficacy and a 23.5% decrease in low self-efficacy. After the intervention, QOL also increased by 38.2%, and HbA_1c_ levels considerably dropped (*P*<.01), but the control group had no significant improvements. These findings demonstrate the model’s ability to improve glycemic management and general health. The FCEM provides patients’ families the tools they need to better understand their lifestyle issues, develop their patient support techniques, and alter their own living conditions [9]. The FCEM can boost a patient’s self-efficacy and self-esteem because it is linked to their self-participation. Studies have demonstrated a strong correlation between patients’ better eating habits and their belief of self-efficacy [22,23]. The data show that family-centered education improved the adherence of patients with T1DM to therapy and HbA_1c_ results. This shows that improving treatment compliance and health indicators may result from incorporating families into the educational process. A typical education program and an empowerment program vary primarily in that the former is a tactic or plan, while the latter is more of a manual for patients and health care personnel [24,25]. To the best of our knowledge, this is the first randomized controlled clinical trial in Jordan to explore the effect of FCEM on QOL, self-efficacy, and HbA_1c_ levels in adolescents with T1DM. The results of this study indicated that most of the teenagers under investigation had poor levels of self-efficacy in their pretransition educational programs. This may be because teenagers are still learning a lot of skills required for self-managing their diabetes and realizing how important it is to have continuous assistance from their families in order to maintain good control of HbA_1c_ level. Our findings were consistent with other research demonstrating the link between strong control of HbA_1c_ level and high levels of self-efficacy. Our study showed that the mean total score of self‐efficacies enhanced better in the intervention group after the intervention compared to that in the control group. This result is consistent with the assessment conducted by Gutierrez-Colina et al [26] on 44 young people with T1DM and discovered that the young adolescent had lower levels of self-efficacy at the baseline evaluation. On the contrary, Survonen et al [27] found that the teenagers’ self-efficacy level was good at the beginning of the assessment. The mean HbA_1c_ level was lower in the intervention group 6 months after the intervention as compared to the control group, as the results showed. In a comparable direction, the findings of the study suggest that boosting problem-solving skills and self-efficacy might help improve self-management, which in turn can improve glycemic control [28]. In addition, another study states that teenagers with T1DM who also have higher levels of self-efficacy are more likely to reach their diabetes management goals [29]. Additionally, this finding is in accordance with research, which showed that HbA_1c_ levels were lowered by patients with diabetes and their families being empowered in home-centered care [30]. The empowerment model’s contribution to greater increases in hemoglobin levels appears to be dependent on family engagement, as evidenced by the comparison of intervention and control groups. It makes sense that those with lower HbA_1c_ levels would follow healthy diets and adjust their eating habits. The results of this study indicate that while young people with T1DM may have difficulties in handling treatment requests from their parents, it might be advantageous for them to have parental participation in order to improve their QOL specifically connected to diabetes. This may be explained by the fact that educational program enhances teenagers’ capacity to their illness and capacity to handle stressful life situations. This result agrees with the study that evaluated the impact of self-reported chronic-generic and condition-specific QOL on glycemic control among adolescents and showed that QOL was inversely associated with HbA_1c_ after 3 years in the course of T1DM only in patients poorly controlled at baseline [31,32]. A significant relationship between glycemic control and QOL was found in a study including 240 Emirati individuals with diabetes. In particular, negative correlations are shown between the HbA_1c_ and each QOL subdomain [33]. Previous research found that there is no discernible relationship between the QOL of adolescents with chronic illnesses and their overall preparedness for the move to adult care [34], while another study highlights the complexity and diversity of factors affecting the transition of experiences and QOL in teenagers with chronic conditions [35], several investigations have pointed to the significant correlation between the total self-efficacy and total QOL at both short- and long-term assessments after the program. In a prior study, Ayar et al [36] found that while the web-based diabetes education program had no effect on A_1c_ levels, it was helpful in raising the self-efficacy and QOL of teenagers with diabetes. Furthermore, they discovered that the intervention group’s self-efficacy levels were higher than those receiving only standard care. Improving self-efficacy in teenagers with T1DM is crucial for encouraging positive changes in their behavior related to optimal self-management. The adolescents diagnosed with T1DM in the intervention group and the control group exhibited notable discrepancies in their QOL mean scores. Statistical analysis revealed a significant difference between the mean QOL scores of the 2 groups. Specifically, the control group demonstrated a lower mean QOL score compared to the intervention group. This suggests that adolescents with T1DM who did not receive the intervention experienced a lower overall QOL compared to those who participated in the intervention program. These findings underscore the potential impact of the intervention in enhancing the QOL among adolescents with T1DM, highlighting the importance of targeted interventions in improving well-being in this population [36]. A study by Nouira et al [37] found that a significant proportion of patients faced academic difficulties, with 71.4% repeating grades and 47.1% discontinuing schooling. Socioeconomic status did not significantly impact academic outcomes. Insulin therapy was more strongly associated with school failure than analogs. Consistent self-monitoring was also linked to academic underachievement. Only 41% of students reached the target HbA_1c_ level.

### Strength and Limitation

The main strength of our study is that it is the first-ever study conducted in Jordan to assess the effect of FCEM on QOL, self-efficacy, and HbA_1c_ levels in adolescents with T1DM. When compared to other study designs, this randomized controlled clinical trial has several advantages. They are, by contrast, easy, quick, and inexpensive. The brief follow-up time is recognized as a research design weakness. The complaint of the follow-up time emphasizes how essential it is for research projects to take the length of the observation into account. A longer follow-up time is frequently required to make more firm conclusions on the long-term impact and efficacy of treatments in health care research, even if short-term outcomes might still provide valuable information. On the other hand, the reasons for longer follow-up are as follows: first, sustainability of outcomes: the investigation has highlighted learning in HbA_1c_, self-efficacy, and QOL gains immediately after the intervention—however, longer-term follow-up would judge if these gains are maintained. Diabetes is a chronic illness, and this study involves T1DM; thus, it is important to consider whether the effects of the intervention persist when those with diabetes move to the next phase of their adolescence while they continue to live with the illness. Second, behavioral changes: diabetes requires some extra behaviors that when implemented may not be possible to cultivate within the shortest period. A longer follow-up could help to answer more questions about whether the participants retain the self-management strategies developed during the actual intervention as well as changes to these behaviors over time. Third, adjustment to life changes: the things that the growing adolescent needs may also change as he or she progresses through the different stage of development. Further follow-up would enable scientists to learn whether and how such an intervention influences changes to these emergent challenges and opportunities to better manage health. Fourth, understanding relapse patterns: a longer follow-up time would be of more importance perhaps to detect any trends in relapse or rather a return to poorer management practices. Understanding why and when these relapses ensue may help fine-tune later interventions and assistance for the varying continued requirements of adolescent individuals and their kin. Fifth, family dynamics: as previously stated, the most significant feature of the approach is to consider the role of family support in regulating T1DM. To give deeper insight into the type of intervention, it would be more appropriate to assess the involvement of the family in supporting the health of the adolescent in the future.

### Implications for Nursing Practice and Policy

This study delves into the implications for nursing and health policy arising from the influence of family-centered empowerment on Jordanian adolescents with T1DM. The primary focus is on the measurable outcomes of HbA_1c_ levels, self-efficacy, and QOL in this specific demographic. By examining these factors, the research aims to contribute valuable insights that can inform nursing practices and health policies to better support adolescents with T1DM in Jordan and also to provide evidence-based insights into the implications of family-centered empowerment on Jordanian adolescents with T1DM. The findings will contribute to the development of targeted nursing interventions and informed health policies, ultimately improving the well-being and outcomes of this specific population.

### Conclusions

This randomized controlled trial suggests that patients with T1DM should receive continuous care education sessions in their regular clinic appointments to evaluate the disease’s long-term consequences. Programs for transitioning to adult care should consider self-efficacy and QOL. Nurses should incorporate self-care training into treatment plans to improve health. Additionally, educational programs should be held for children and adolescents to raise awareness and adherence to diabetes care recommendations, ultimately improving the QOL for all patients with diabetes.

## Appendix

Multimedia Appendix 1 CONSORT (Consolidated Standards of Reporting Trials) 2010 checklist.

Multimedia Appendix 2 Demographic data questionnaire.

Multimedia Appendix 3 Self-Efficacy Questionnaire for Children.

Multimedia Appendix 4 The Pediatric Quality of Life Inventory 3.0 Diabetes Module.

Multimedia Appendix 5 The data file of the study.

Multimedia Appendix 6 Consent form.

## Abbreviations

CONSORT: Consolidated Standards of Reporting Trials
FCEM: family-centered empowerment model
HbA1c: glycosylated hemoglobin
QOL: quality of life
SEQ: Self-Efficacy Questionnaire
T1DM: type 1 diabetes mellitus

## Data Availability

All data generated or analyzed during this study are included in this published paper and in Multimedia Appendix 5.
