# Supplementary material for: Improving Self-Efficacy, Quality of Life, and Glycemic Control in Adolescents With Type 1 Diabetes: Randomized Controlled Trial for the Evaluation of the Family-Centered Empowerment Model
Source: JMIR Form Res. 2024 Dec 10;8:e64463. doi: 10.2196/64463 (PMC11668983; doi:10.2196/64463)
Supplement: Multimedia Appendix 2 [file formative_v8i1e64463_app2.pdf]

Demographic Data Questionnaire
(English Version)

Date: .... **Participant Code Number:** ....

Demographic Data Questionnaire

❖ Who is answering the general questions of this questionnaire?

☐ The Participant ☐ His Father ☐ His Mother ☐ Other (Please specify........)

- Please answer each question as accurately as possible by circling the correct answer or filling in the space provided.

1. What is your age? ___________________

2. What is your gender? ☐ Female ☐ Male

3. Are you currently a student? ☐ Yes ☐ No

▪ If (Yes), what is your grade? ___________________

4. Parents marital status: ☐ Married ☐ Divorced

5. Do you live with both of them? ☐ Yes ☐ No

▪ If (No), what is the reason? ☐Death ☐Divorced ☐Father's travel abroad

6. What is the highest level of education your father has completed?

☐ Less than high school degree
☐ High school degree or equivalent
☐ College degree
☐ Bachelor degree
☐ Master degree
☐ Doctoral degree

7. What is the highest level of education your mother has completed?

☐ Less than high school degree
☐ High school degree or equivalent
☐ College degree
☐ Bachelor degree
☐ Master degree
☐ Doctoral degree

8. What is your father employment status?

☐ Unemployed
☐ Part-time
☐ Full-time

9. What is your mother employment status?

- ☐ Unemployed
- ☐ Part-time
- ☐ Full-time

10. For how long you have been diagnosed with diabetes?

☐1 - <2 years ☐2 - <3 years ☐3 - <4 years ☐4 - <5 years ☐5 years or more

11. Age at diagnosis: ____________________

12. Has any of your family members diagnosed with diabetes? ☐ Yes ☐ No

- If yes, who:

☐ Father ☐ Mother ☐ Brother ☐ Sister

13. What other medical conditions or diseases do you have other than type 1diabetes?

- ☐ Cystic fibrosis
- ☐ Asthma
- ☐ Cardiovascular disease
- ☐ Kidney diseases
- ☐ Autoimmune disease (Please specify ....)
- ☐ Eating disorder
- ☐ Depression
- ☐ Other (Please specify ....)
- ☐ Don't know

14. What is your height?____________________cm

15. What is your weight?____________________kg

16. What is the mode of insulin delivery? ☐ Daily insulin injections ☐ Insulin pump

- If on Daily injections, number of injections per day
- How often is your blood sugar measured per day?

17. Do you have health insurance coverage? ☐ Yes ☐ No

18. How many times have you been hospitalized due to diabetes last year?

19. How many episodes of hypoglycemia have you reported last month?

Thank you for completing this personal profile

IRB Number: NEU/2023/110-1681

IRB Approval Date: 26/01/2023

إستبيان المعلومات الشخصية

- **من يجيب على الأسئلة العامة لهذا الاستبيان؟**

المشارك ☐ الأب ☐ الأم ☐ أحد أخر (الرجاء التحديد ....)

- **يرجى الإجابة على كل سؤال بأكبر قدر ممكن من الدقة عن طريق إختيار الإجابة الصحيحة أو ملء المساحة المتوفرة.**

1. **العمر** .... سنة
2. **الجنس** ☐ ذكر ☐ أنثى
3. **هل أنت ملتحق بالمدرسة حاليا؟** ☐ نعم ☐ لا
   - إذا كانت الإجابة ب (**نعم**), الرجاء تحديد مستوى الصف الدراسي ....
4. **الحالة الإجتماعية للأبوين:** ☐ متزوجيين ☐ مطلقين
5. **هل تعيش مع كليهما:** ☐ نعم ☐ لا
   - إذا كانت الإجابة ب (**لا**), فما هو السبب؟ ☐ الطلاق ☐ الوفاة ☐ سفر الأب الى الخارج
6. **ما هو المستوى التعليمي للأب؟**
   - ☐ أقل من الثانوية العامة
   - ☐ الثانوية العامة أو ما يعادلها
   - ☐ دبلوم كلّية
   - ☐ درجة جامعية- بكالوريوس
   - ☐ درجة جامعية- ماجستير
   - ☐ درجة جامعية- دكتوراة

7. ما هي طبيعة عمل الأب؟

- ☐ بدون عمل
- ☐ وظيفة بدوام جزيء
- ☐ وظيفة بدوام كامل

8. ما هو المستوى التعليمي للأم؟

- ☐ أقل من الثانوية العامة
- ☐ الثانوية العامة أو ما يعادلها
- ☐ دبلوم كلّية
- ☐ درجة جامعية- بكالوريوس
- ☐ درجة جامعية- ماجستير
- ☐ درجة جامعية- دكتوراة

9. ما هي طبيعة عمل الأم؟

- ☐ بدون عمل
- ☐ وظيفة بدوام جزيء
- ☐ وظيفة بدوام كامل

10. منذ متى تم تشخيصك بمرض السكري؟

- ☐ سنة إلى أقل من سنتين
- ☐ سنتين إلى أقل من 3 سنوات
- ☐ 3 سنوات إلى أقل من 4 سنوات
- ☐ 4 سنوات إلى أقل من 5 سنوات
- ☐ 5 سنوات أو أكثر

11. العمر عند التشخيص: .... سنة

12. هل هناك أحد من أفراد عائلتك مصاب بالسكري؟ ☐ نعم ☐ لا

♦ إذا كانت الإجابة ب (نعم), الرجاء الإشارة ب (√) على الشخص:

☐ الأب ☐ الأم ☐ الأخ ☐ الأخت

13. هل تعاني من أي من الأمراض التالية بالإضافه لمرض السكري؟

☐ التليّف الكيسي

☐ الربو

☐ أمراض القلب و الشرايين

☐ أمراض الكلى

☐ أمراض المناعة (الرجاء التحديد.......................................)

☐ إضطرابات الأكل

☐ الأكتئاب

☐ أمراض أخرى (الرجاء التحديد.......................................)

☐ لا أعرف

14. الطول: .... سم

15. الورن: .... كغم

16. ما هي الكيفية التي تتناول بها جرعة الأنسولين؟

☐ حُقن الإنسولين اليومية (الحقن تحت الجلد)

☐ جهاز مضخة الأنسولين

♦ إذا كانت الإجابة ب (حُقن الإنسولين اليومية), كم هو عدد الحُقن يومياً....................

♦ كم مرة يتم قياس نسبة السكر في الدم يومياً؟ ....

17. هل لديك تأمين صحي؟ ☐ نعم ☐ لا

18. كم هي عدد المرات التي كنت قد أدخلت بها المستشفى بسبب مرض السكري في العام الماضي؟
....

19. كم هي عدد الحالات التي عانيت فيها من نقص السكر في الدم الشهر الماضي؟
....

شكراً على إكمال هذا الإستبيان

IRB Number: NEU/2023/110-1681

IRB Approval Date: 26/01/2023
