# Supplementary material for: Improving Self-Efficacy, Quality of Life, and Glycemic Control in Adolescents With Type 1 Diabetes: Randomized Controlled Trial for the Evaluation of the Family-Centered Empowerment Model
Source: JMIR Form Res. 2024 Dec 10;8:e64463. doi: 10.2196/64463 (PMC11668983; doi:10.2196/64463)
Supplement: Multimedia Appendix 3 [file formative_v8i1e64463_app3.pdf]

Self-Efficacy Questionnaire for Children (SEQ-C)

Self-Efficacy Questionnaire for Children (SEQ-C)

	1<br>Not at all	2	3	4	5<br>Very well
1. How well can you get teachers to help you when you get stuck on schoolwork?	○	○	○	○	○
2. How well can you express your opinions when other classmates disagree with you?	○	○	○	○	○
3. How well do you succeed in cheering yourself up when an unpleasant event has happened?	○	○	○	○	○
4. How well can you study when there are other interesting things to do?	○	○	○	○	○
5. How well do you succeed in becoming calm again when you are very scared?	○	○	○	○	○
6. How well can you become friends with other children?	○	○	○	○	○
7. How well can you study a chapter for a test?	○	○	○	○	○
8. How well can you have a chat with an unfamiliar person?	○	○	○	○	○
9. How well can you prevent to become nervous?	○	○	○	○	○
10. How well do you succeed in finishing all your homework every day?	○	○	○	○	○
11. How well can you work in harmony with your classmates?	○	○	○	○	○
12. How well can you control your feelings?	○	○	○	○	○
13. How well can you pay attention during every class?	○	○	○	○	○
14. How well can you tell other children that they are doing something that you don’t like?	○	○	○	○	○
15. How well can you give yourself a pep-talk when you feel low?	○	○	○	○	○
16. How well do you succeed in understanding all subjects in school?	○	○	○	○	○
17. How well can you tell a funny event to a group of children?	○	○	○	○	○
18. How well can you tell a friend that you don’t feel well?	○	○	○	○	○
19. How well do you succeed in satisfying your parents with your schoolwork?	○	○	○	○	○
20. How well do you succeed in staying friends with other children?	○	○	○	○	○
21. How well do you succeed in suppressing unpleasant thoughts?	○	○	○	○	○
22. How well do you succeed in passing a test?	○	○	○	○	○
23. How well do you succeed in preventing quarrels with other children?	○	○	○	○	○
24. How well do you succeed in not worrying about things that might happen?	○	○	○	○	○

Scoring

A total self-efficacy score can be obtained by summing across all items.
Items 1, 4, 7, 10, 13, 16, 19, and 22 = Academic self-efficacy

Items 2, 6, 8, 11, 14, 17, 20, and 23 = Social self-efficacy
Items 3, 5, 9, 12, 15, 18, 21, and 24 = Emotional self-efficacy

Key references

Muris, P. (2001). A brief questionnaire for measuring self-efficacy in youths. *Journal of Psychopathology and Behavioral Assessment*, *23*, 145-149.

Muris, P. (2002). Relationships between self-efficacy and symptoms of anxiety disorders and depression in a normal adolescent sample. *Personality and Individual Differences*, *32*, 337-348

Note

Three items of this questionnaire were taken from Bandura et al. (1999). See: Bandura, A., Pastorelli, C., Barbaranelli, C., & Caprara, G.V. (1999). Self-efficacy pathways to childhood depression. *Journal of Personality and Social Psychology*, *76*, 258-269.

استبيان الكفاءة الذاتية للأطفال

1. ما مدى جودة مساعدة المعلمين لك عندما تتعثر في العمل المدرسي؟

5	4	3	2	1
**ممتاز**				**لا على الإطلاق**
○	○	○	○	○

2. إلى أي مدى يمكنك التعبير عن آرائك بشكل جيد عندما يختلف معك زملائك في الفصل؟

○ ○ ○ ○ ○

3. ما مدى نجاحك في ابتهاج نفسك عندما يقع حدث غير سار؟

○ ○ ○ ○ ○

4. ما مدى جودة المذاكرة عندما تكون هناك أشياء أخرى ممتعة يمكنك القيام بها؟

○ ○ ○ ○ ○

5. ما مدى نجاحك في أن تصبح هادئًا مرة أخرى عندما تكون خائفًا جدًا؟

○ ○ ○ ○ ○

6. ما مدى قدرتك على تكوين صداقات مع أطفال آخرين؟

○ ○ ○ ○ ○

7. ما مدى جودة دراسة فصل للاختبار؟

○ ○ ○ ○ ○

8. إلى أي مدى يمكنك إجراء محادثة مع شخص غير مألوف؟

○ ○ ○ ○ ○

9. إلى أي مدى يمكنك منع أن تصبح عصبيًا؟

○ ○ ○ ○ ○

10. ما مدى نجاحك في إنهاء جميع واجباتك المدرسية كل يوم؟

○ ○ ○ ○ ○

11. إلى أي مدى يمكنك العمل بانسجام مع زملائك في الفصل؟

○ ○ ○ ○ ○

12. إلى أي مدى يمكنك التحكم في مشاعرك؟

○ ○ ○ ○ ○

13. إلى أي مدى يمكنك الانتباه جيدًا خلال كل فصل؟

○ ○ ○ ○ ○

14. إلى أي مدى يمكنك إخبار الأطفال الآخرين بأنهم يفعلون شيئًا لا تحبه؟

○ ○ ○ ○ ○

15. إلى أي مدى يمكنك التحدث مع نفسك عندما تشعر بالإحباط؟

○ ○ ○ ○ ○

16. ما مدى نجاحك في فهم جميع المواد في المدرسة؟

○ ○ ○ ○ ○

17. إلى أي مدى يمكنك إخبار حدث مضحك لمجموعة من الأطفال؟

○ ○ ○ ○ ○

18. إلى أي مدى يمكنك إخبار صديق أنك لست على ما يرام؟

○ ○ ○ ○ ○

19. ما مدى نجاحك في إرضاء والديك بواجباتك المدرسية؟

○ ○ ○ ○ ○

20. ما مدى نجاحك في البقاء صديقًا للأطفال الآخرين؟

○ ○ ○ ○ ○

21. ما مدى نجاحك في قمع الأفكار غير السارة؟

○ ○ ○ ○ ○

22. ما مدى نجاحك في اجتياز الاختبار؟

○ ○ ○ ○ ○

23. ما مدى نجاحك في منع الخلافات مع الأطفال الآخرين؟

○ ○ ○ ○ ○

24. ما مدى نجاحك في عدم القلق بشأن الأشياء التي قد تحدث؟

○ ○ ○ ○ ○

حساب النتيجة

يمكن الحصول على مجموع نقاط الكفاءة الذاتية من خلال جمع جميع العناصر.

البنود 1 و 4 و 7 و 10 و 13 و 16 و 19 و 22 = **الكفاءة الذاتية الأكاديمية**

البنود 2 و 6 و 8 و 11 و 14 و 17 و 20 و 23 = **الكفاءة الذاتية الاجتماعية**

البنود 3 و 5 و 9 و 12 و 15 و 18 و 21 و 24 = **الكفاءة الذاتية العاطفية**
