# Supplementary material for: Improving Self-Efficacy, Quality of Life, and Glycemic Control in Adolescents With Type 1 Diabetes: Randomized Controlled Trial for the Evaluation of the Family-Centered Empowerment Model
Source: JMIR Form Res. 2024 Dec 10;8:e64463. doi: 10.2196/64463 (PMC11668983; doi:10.2196/64463)
Supplement: Multimedia Appendix 4 [file formative_v8i1e64463_app4.pdf]

The Pediatric Quality of Life Inventory
3.0 Diabetes Module – English Version

PedsQL ™

Diabetes Module

Version 3.0

TEEN REPORT (ages **13-18**)

DIRECTIONS

Teens with diabetes sometimes have special problems. Please tell us **how much of a problem** each one has been for you during the **past ONE month** by circling:

0 if it is **never** a problem
1 if it is **almost never** a problem
2 if it is **sometimes** a problem
3 if it is **often** a problem
4 if it is **almost always** a problem

There are no right or wrong answers.
If you do not understand a question, please ask for help.

Not to be reproduced without permission

Copyright © 1998 JW Varni, Ph.D.
All rights reserved

UTHealth The University of Texas Health Science Center at Houston
IRB NUMBER: HSC-SN-17-0852
IRB APPROVAL DATE: 10/10/2017

Date: ………………………………… **Participant Code Number:** …………………………………………………………

*In the past **ONE month,** how much of a **problem** has this been for you …*

**ABOUT MY DIABETES** ***(problems with…)***	Never	Almost Never	Some-times	Often	Almost Always
1. I feel hungry	0	1	2	3	4
2. I feel thirsty	0	1	2	3	4
3. I have to go to the bathroom too often	0	1	2	3	4
4. I have stomachaches	0	1	2	3	4
5. I have headaches	0	1	2	3	4
6. I go “low”	0	1	2	3	4
7. I feel tired or fatigued	0	1	2	3	4
8. I get shaky	0	1	2	3	4
9. I get sweaty	0	1	2	3	4
10. I have trouble sleeping	0	1	2	3	4
11. I get irritable	0	1	2	3	4

**TREATMENT - I** ***(problems with…)***	Never	Almost Never	Some-times	Often	Almost Always
1. It hurts to prick my finger or give insulin shots	0	1	2	3	4
2. I am embarrassed about having diabetes	0	1	2	3	4
3. My parents and I argue about my diabetes care	0	1	2	3	4
4. It is hard for me to stick to my diabetes care plan	0	1	2	3	4

*Whether you do these things **on your own or with the help of your parents**, please answer how hard these things were to do in the past **ONE month**.*

**TREATMENT II -** ***(problems with…)***	Never	Almost Never	Some-times	Often	Almost Always
1. It is hard for me to take blood glucose tests	0	1	2	3	4
2. It is hard for me to take insulin shots	0	1	2	3	4
3. It is hard for me to exercise	0	1	2	3	4
4. It is hard for me to keep track of carbohydrates or exchanges	0	1	2	3	4
5. It is hard for me to wear my id bracelet	0	1	2	3	4
6. It is hard for me to carry a fast-acting carbohydrate	0	1	2	3	4
7. It is hard for me to eat snacks	0	1	2	3	4

**WORRY** ***(problems with…)***	Never	Almost Never	Some-times	Often	Almost Always
1. I worry about “going low”	0	1	2	3	4
2. I worry about whether or not my medical treatments are working	0	1	2	3	4
3. I worry about long-term complications from diabetes	0	1	2	3	4

Not to be reproduced without permission

UTHealth The University of Texas Health Science Center at Houston
IRB NUMBER: HSC-SN-17-0852
IRB APPROVAL DATE: [illegible]/2017
Copyright © 1998 JW Varni, Ph.D. [illegible]ghts reserved

Date: .... **Participant Code Number:** ....

PedsQL 3

*In the past **ONE month,** how much of a **problem** has this been for you …*

COMMUNICATION *(problems with…)*	Never	Almost Never	Some-times	Often	Almost Always
1. It is hard for me to tell the doctors and nurses how I feel	0	1	2	3	4
2. It is hard for me to ask the doctors and nurses questions	0	1	2	3	4
3. It is hard for me to explain my illness to other people	0	1	2	3	4

Not to be reproduced without permission

UTHealth The University of Texas Health Science Center at Houston

IRB NUMBER: HSC-SN-17-0852
IRB APPROVAL DATE: 10/10/2017

Copyright © 1998 JW Varni, Ph.D. [illegible]ghts reserved

The Pediatric Quality of Life Inventory

3.0 Diabetes Module – Arabic Version

رقم البطاقة الشخصية_____________

التاريخ:_________________

PedsQL™

استطلاع مرض السكر

Version 3.0 - Arabic (Jordan)

تقرير الأطفال (للأعمار **8-12**)

التوجيهات

في بعض الأحيان، يواجه الأطفال الذين يعانون من مرض السكر مشاكل ذات طابع خاص.

0 إذا كان لا يمثل مشكلة **أبدًا**
1 إذا كان **نادرًا** ما يمثل مشكلة
2 إذا كان **أحيانًا** ما يمثل مشكلة
3 إذا كان **غالبًا** ما يمثل مشكلة
4 إذا كان **دائمًا تقريبًا** ما يمثل مشكلة

ليست هناك إجابات صحيحة أو خاطئة.
إذا كنت لا تفهم/تفهمي سؤالاً، فمن فضلك اطلب/اطلبي المساعدة.

JW Varni, Ph.D. 1998 © حقوق التأليف والنشر
جميع الحقوق محفوظة
لا يُسمح بإعادة إصداره بدون إذن

IRB NUMBER: HSC-SN-17-0892 00/09
UTHealth The University of Texas IRB APPROVAL DATE: 10/10/2017
PedsQL – Jordan/Arabic – Version of 11 Jun 12 – MAPI Institute.
ID6710 / PedsQL-3.0-Diabetes-C_AU3.0_ara-JO.doc

*خلال **الـ 4 أسابيع** الماضية، ما مدى **المشكلة** التي كان يمثلها لك ذلك ...*

**عن مرض السكر** *(مشكلات مع...)*	أبدًا	نادرًا	أحيانًا	غالبًا	دائمًا
1. أشعر بالجوع	0	1	2	3	4
2. أشعر بالعطش	0	1	2	3	4
3. يجب أن أذهب إلى الحمام مرات كثيرة جدًا	0	1	2	3	4
4. عندي مغص	0	1	2	3	4
5. عندي صداع	0	1	2	3	4
6. "ينخفض مستوى السكر في دمي"	0	1	2	3	4
7. أشعر بالتعب	0	1	2	3	4
8. أشعر برعشة	0	1	2	3	4
9. أتبلّل من العرق	0	1	2	3	4
10. لا أنام جيدًا	0	1	2	3	4
11. أصبحت أنفعل بسرعة	0	1	2	3	4

**العلاج - 1** *(مشكلات مع...)*	أبدًا	نادرًا	أحيانًا	غالبًا	دائمًا
1. أتألم عند وخز إصبعي أو إعطاء حقن الإنسولين	0	1	2	3	4
2. أشعر بالحرج من مرض السكر	0	1	2	3	4
3. أتجادل أنا ووالديّ حول الرعاية الخاصة بمرض السكر	0	1	2	3	4
4. من الصعب عليّ أن ألتزم بخطة الرعاية الخاصة بمرض السكر	0	1	2	3	4

*سواء كنت تفعل/تفعلي هذه الأشياء **وحدك أو بمساعدة والديك**، من فضلك قُل/قولي مدى صعوبة قيامك بهذه الأشياء خلال **الـ 4 أسابيع** الماضية.*

**العلاج - 2** *(مشكلات مع...)*	أبدًا	نادرًا	أحيانًا	غالبًا	دائمًا
1. من الصعب عليّ إجراء اختبار السكر في الدم	0	1	2	3	4
2. من الصعب عليّ أخذ حقن الإنسولين	0	1	2	3	4
3. من الصعب عليّ ممارسة الأنشطة البدنية	0	1	2	3	4
4. من الصعب عليّ مراقبة كمية الكربوهيدرات أو البدائل	0	1	2	3	4
5. من الصعب عليّ ارتداء سوار بيانات مرض السكر	0	1	2	3	4
6. من الصعب عليّ حمل كربوهيدرات سريعة المفعول معي	0	1	2	3	4
7. من الصعب عليّ تناول الوجبات الخفيفة	0	1	2	3	4

**القلق** *(مشكلات مع...)*	أبدًا	نادرًا	أحيانًا	غالبًا	دائمًا
1. أقلق أن "ينخفض مستوى السكر في دمي"	0	1	2	3	4
2. أقلق حول ما إذا كانت العلاجات الطبية التي أتلقاها ناجحة أم لا	0	1	2	3	4
3. أقلق من المضاعفات طويلة المدى الناتجة عن مرض السكر	0	1	2	3	4

لا يُسمح بإعادة إصداره بدون إذن
حقوق التأليف والنشر © 1998 JW Varni, Ph.D.
جميع الحقوق محفوظة
PedsQL – Jordan/Arabic – Version of 11 Jun 12 – MAPI Institute.
ID6710 / PedsQL-3.0-Diabetes-C_AU3.0_ara-JO.doc
UTHealth The University of Texas Health Science Center at Houston
IRB NUMBER: HSC-SN-17-0852
IRB APPROVAL DATE: 10/10/2017

*خلال الـ **4 أسابيع** الماضية، ما مدى **المشكلة** التي كان يمثلها لك ذلك ...*

التواصل*(مشكلات مع...)*	أبدًا	نادرًا	أحيانًا	غالبًا	دائمًا
1. من الصعب عليّ أن أقول للأطباء والممرضين ما أشعر به	0	1	2	3	4
2. من الصعب عليّ أن أطرح أسئلة على الأطباء والممرضين	0	1	2	3	4
3. من الصعب عليّ أن أشرح مرضي للآخرين	0	1	2	3	4

لا يُسمح بإعادة إصداره بدون إذن
JW Varni, Ph.D. 1998 © حقوق التأليف والنشر
جميع الحقوق محفوظة
PedsQL – Jordan/Arabic – Version of 11 Jun 12 – MAPI Institute.
ID6710 / PedsQL-3.0-Diabetes-C_AU3.0_ara-JO.doc
UTHealth The University of Texas Health Science Center at Houston
IRB NUMBER: HSC-SN-17-0852
IRB APPROVAL DATE: 10/10/2017

رقم البطاقة الشخصية____________

التاريخ:________________________

PedsQL™

استطلاع مرض السكر

Version 3.0 - Arabic (Jordan)

تقرير المراهقين (للأعمار **13-18**)

التوجيهات

في بعض الأحيان، يواجه المراهقون الذين يعانون من مرض السكر مشاكل ذات طابع

0 إذا كان لا يمثل مشكلة **أبدًا**
1 إذا كان **نادرًا** ما يمثل مشكلة
2 إذا كان **أحيانًا** ما يمثل مشكلة
3 إذا كان **غالبًا** ما يمثل مشكلة
4 إذا كان **دائمًا تقريبًا** ما يمثل مشكلة

ليست هناك إجابات صحيحة أو خاطئة.
إذا كنت لا تفهم/تفهمي سؤالاً، فمن فضلك اطلب/اطلبي المساعدة.

حقوق التأليف والنشر © 1998 JW Varni, Ph.D.
جميع الحقوق محفوظة
لا يُسمح بإعادة إصداره بدون إذن

IRB NUMBER: HSC-SN-17-0852
UTHealth The University of Texas Health Science Center at Houston IRB APPROVAL DATE: 10/10/2017
PedsQL – Jordan/Arabic – Version of 11 Jun 12 – MAPI Institute.
ID6710 / PedsQL-3.0-Diabetes-A_AU3.0_ara-JO.doc

*خلال **الـ 4 أسابيع** الماضية، ما مدى **المشكلة** التي كان يمثلها لك ذلك ...*

**عن مرض السكر** *(مشكلات مع...)*	أبدًا	نادرًا	أحيانًا	غالبًا	دائمًا
1. أشعر بالجوع	0	1	2	3	4
2. أشعر بالعطش	0	1	2	3	4
3. يجب أن أذهب إلى الحمام مرات كثيرة جدًا	0	1	2	3	4
4. عندي مغص	0	1	2	3	4
5. عندي صداع	0	1	2	3	4
6. "ينخفض مستوى السكر في دمي"	0	1	2	3	4
7. أشعر بالتعب	0	1	2	3	4
8. أشعر برعشة	0	1	2	3	4
9. أتبلّل من العرق	0	1	2	3	4
10. لا أنام جيدًا	0	1	2	3	4
11. أصبحت أنفعل بسرعة	0	1	2	3	4

**العلاج - 1** *(مشكلات مع...)*	أبدًا	نادرًا	أحيانًا	غالبًا	دائمًا
1. أتألم عند وخز إصبعي أو إعطاء حقن الإنسولين	0	1	2	3	4
2. أشعر بالحرج من مرض السكر	0	1	2	3	4
3. أتجادل أنا ووالديّ حول الرعاية الخاصة بمرض السكر	0	1	2	3	4
4. من الصعب عليّ أن ألتزم بخطة الرعاية الخاصة بمرض السكر	0	1	2	3	4

*سواء كنت تفعل/تفعلي هذه الأشياء **وحدك أو بمساعدة والديك**، من فضلك قُل/قولي مدى صعوبة قيامك بهذه الأشياء خلال **الـ 4 أسابيع** الماضية.*

**العلاج - 2** *(مشكلات مع...)*	أبدًا	نادرًا	أحيانًا	غالبًا	دائمًا
1. من الصعب عليّ إجراء اختبار السكر في الدم	0	1	2	3	4
2. من الصعب عليّ أخذ حقن الإنسولين	0	1	2	3	4
3. من الصعب عليّ ممارسة الأنشطة البدنية	0	1	2	3	4
4. من الصعب عليّ مراقبة كمية الكربوهيدرات أو البدائل	0	1	2	3	4
5. من الصعب عليّ ارتداء سوار بيانات مرض السكر	0	1	2	3	4
6. من الصعب عليّ حمل كربوهيدرات سريعة المفعول معي	0	1	2	3	4
7. من الصعب عليّ تناول الوجبات الخفيفة	0	1	2	3	4

**القلق** *(مشكلات مع...)*	أبدًا	نادرًا	أحيانًا	غالبًا	دائمًا
1. أقلق أن "ينخفض مستوى السكر في دمي"	0	1	2	3	4
2. أقلق حول ما إذا كانت العلاجات الطبية التي أتلقاها ناجحة أم لا	0	1	2	3	4
3. أقلق من المضاعفات طويلة المدى الناتجة عن مرض السكر	0	1	2	3	4

لا يُسمح بإعادة إصداره بدون إذن
حقوق التأليف والنشر © JW Varni, Ph.D. 1998
جميع الحقوق محفوظة
IRB NUMBER: HSC-SN-17-0852
UTHealth The University of Texas Health Science Center at Houston
IRB APPROVAL DATE: 10/10/2017
PedsQL – Jordan/Arabic – Version of 11 Jun 12 – MAPI Institute.
ID6710 / PedsQL-3.0-Diabetes-A_AU3.0_ara-JO.doc

*خلال **الـ 4 أسابيع** الماضية، ما مدى **المشكلة** التي كان يمثلها لك ذلك ...*

**التواصل** *(مشكلات مع...)*	أبدًا	نادرًا	أحيانًا	غالبًا	دائمًا
1. من الصعب عليّ أن أقول للأطباء والممرضين ما أشعر به	0	1	2	3	4
2. من الصعب عليّ أن أطرح أسئلة على الأطباء والممرضين	0	1	2	3	4
3. من الصعب عليّ أن أشرح مرضي للآخرين	0	1	2	3	4

حقوق التأليف والنشر © JW Varni, Ph.D. 1998
جميع الحقوق محفوظة
لا يُسمح بإعادة إصداره بدون إذن

PedsQL – Jordan/Arabic – Version of 11 Jun 12 – MAPI Institute.
ID6710 / PedsQL-3.0-Diabetes-A_AU3.0_ara-JO.doc
UTHealth The University of Texas Health Science Center at Houston
IRB NUMBER: HSC-SN-17-0852
IRB APPROVAL DATE: 10/10/2017
