# Supplementary material for: Improving Self-Efficacy, Quality of Life, and Glycemic Control in Adolescents With Type 1 Diabetes: Randomized Controlled Trial for the Evaluation of the Family-Centered Empowerment Model
Source: JMIR Form Res. 2024 Dec 10;8:e64463. doi: 10.2196/64463 (PMC11668983; doi:10.2196/64463)
Supplement: Multimedia Appendix 6 [file formative_v8i1e64463_app6.pdf]

استمارة موافقة المريض على المشاركة في اجراء بحث علمي

التاريخ: ....

رقم كود المشارك: ....

اسم المريض: ....

الرقم الوطني للمريض: ....

عمر المريض: ....

اسم الباحث: صلاح عبدالله الزواهره

رقم الهاتف: ....

رقم تلفون العائلة.........................

أخر قراءة للسكر التراكمي: ....

عنوان الدراسة: تأثير نموذج التمكين المعتمد على الأسرة على جودة الحياة والكفاءة الذاتية ومستويات السكر التراكمي، لدى المراهقين المصابين بداء السكري من النوع الأول في عمان ، الأردن.

قبل أن أوافق على المشاركة في البحث ، أبلغني الباحث بما يلي:

1. موافقة الجهات المختصة بالمستشفى على إجراء الدراسة.
2. أهداف الدراسة وإجراءاتها.
3. أي مخاطر محتملة ومتوقعة وأي إزعاج أو فوائد تنشأ عن الدراسة.
4. أي إجراءات أو علاجات بديلة أو محتملة.
5. مخاطر محتملة غير متوقعة.
6. أي تعويض أو علاج طبي مؤمن عليه في حالة حدوث ضرر أو ضرر نتيجة الدراسة.
7. مدة الدراسة.
8. إجراءات سرية البيانات.
9. الحالات التي قد تدفع الباحث إلى منعي من المشاركة في الدراسة.
10. أي جهد إضافي يمكن أن أبذله للدراسة.
11. ماذا يحدث إذا قررت الانسحاب من الدراسة.

12. متى يجب إعلامي بالاستنتاجات الجديدة التي قد تؤثر على قراري للمشاركة في الدراسة.

- إذا كانت لديك أسئلة حول حقوقك كمشارك في هذه الدراسة أو ما يجب عليك فعله إذا تعرضت للأذى ، فيمكنك الاتصال في أي وقت:

الاسم: ...صلاح عبدالله الزواهره...

الهاتف ...0772260125.................................

- مشاركتك في هذه الدراسة طوعية واختيارية. لن يتم معاقبتك أو فقدان أي مزايا إذا قررت عدم المشاركة أو الانسحاب من الدراسة في أي وقت.
- بمجرد التوقيع على هذه الوثيقة ، فإنك تقر بموافقتك على المشاركة طواعية في هذه الدراسة وأن المعلومات الواردة أعلاه موضحة بالكامل.

التاريخ:	اسم المشارك:	توقيعه: ....
التاريخ:	اسم الشاهد الاول:	توقيعه: ....
التاريخ:	اسم الشاهد الثاني:	توقيعه: ....
التاريخ:	اسم المشرف على البحث:	توقيعه: ....

IRB Number: NEU/2023/110-1681

IRB Approval Date: 26/01/2023
